# Oligodendroglial GABAergic Signaling: More Than Inhibition!

**DOI:** 10.1007/s12264-021-00693-w

**Published:** 2021-04-29

**Authors:** Xianshu Bai, Frank Kirchhoff, Anja Scheller

**Affiliations:** Molecular Physiology, Center for Integrative Physiology and Molecular Medicine (CIPMM), University of Saarland, 66421 Homburg, Germany

**Keywords:** GABA, GABA_A_ receptor, GABA_B_ receptor, OPC, Oligodendrocyte lineage

## Abstract

GABA is the main inhibitory neurotransmitter in the CNS acting at two distinct types of receptor: ligand-gated ionotropic GABA_A_ receptors and G protein-coupled metabotropic GABA_B_ receptors, thus mediating fast and slow inhibition of excitability at central synapses. GABAergic signal transmission has been intensively studied in neurons in contrast to oligodendrocytes and their precursors (OPCs), although the latter express both types of GABA receptor. Recent studies focusing on interneuron myelination and interneuron-OPC synapses have shed light on the importance of GABA signaling in the oligodendrocyte lineage. In this review, we start with a short summary on GABA itself and neuronal GABAergic signaling. Then, we elaborate on the physiological role of GABA receptors within the oligodendrocyte lineage and conclude with a description of these receptors as putative targets in treatments of CNS diseases.

## Introduction

GABA (γ-aminobutyric acid), besides glycine, is the main inhibitory neurotransmitter in the central nervous system (CNS) [1]. The existence of GABA in the brain was first detected in 1950 [2], without knowing its biological function. Seven years later, studies found that GABA was the “I factor”, the inhibitory neurotransmitter of the mammalian CNS [3]. Thereafter, GABA and GABAergic signaling on neurons were extensively studied [1]. GABA binds to two classes of receptor in the CNS, GABA_A_ and GABA_B_ receptors, and exerts fast or slow inhibition at synaptic terminals. Decades later, since 1978 [4], glial GABA signaling started to attract interest and is now a major research focus while new roles of glial cells are emerging. Oligodendrocytes (OLs) are the myelinating cells of CNS making them indispensable for fast and efficient action potential conduction. They differentiate from precursor cells (OPCs) [5–8]. Despite lifelong ongoing differentiation into OLs, OPCs maintain a certain cell density due to continuous self-renewal [9–12]. Proliferation and differentiation of OPCs are modulated by growth factors [13–15], as well as by communication between OPCs and axons [16–18]. OPCs are the only glial cells receiving direct synaptic input mediated by glutamate and GABA from excitatory and inhibitory synapses, respectively [17, 19–23]. Furthermore, the myelination of interneurons by mature OLs appears to be a direct consequence of GABA-based interneuron-OPC communication [24–26].

## GABA Synthesis, Release, and Uptake in the Brain

**Fig. 1:**
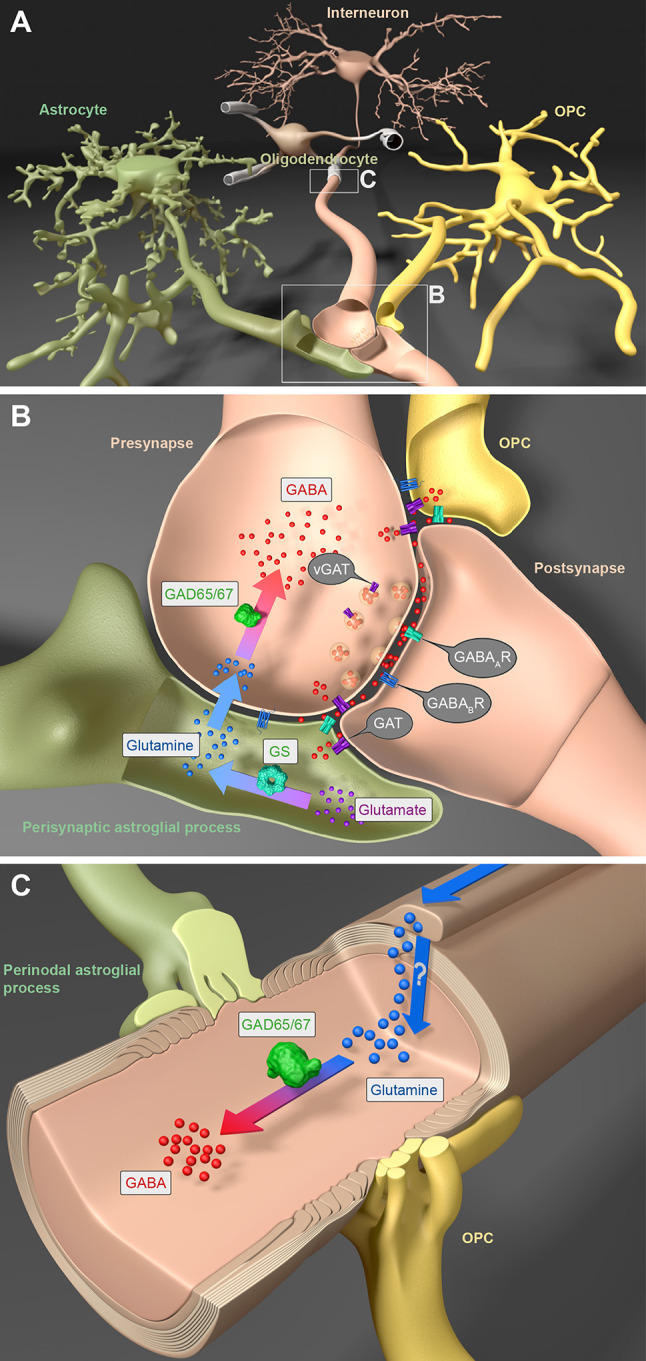
GABA cycling between interneurons, cells of the oligodendrocyte (OL) lineage, and astrocytes. **A** In the central nervous system, interneurons form an intricate signaling network with cells of the OL lineage, i.e., myelinating OLs and their precursors (OPCs), and with perisynaptic as well as perinodal processes of astrocytes. **B** In the synaptic microenvironment, extracellular glutamate is converted into glutamine in astrocytes by glutamine synthetase (GS). After release, glutamine is taken up by interneurons and transformed into GABA by the glutamate decarboxylases GAD65 and/or GAD67. Upon action potential arrival, GABA is released into the synaptic cleft by vesicles expressing GABA transporters (vGAT). After binding to postsynaptic neuronal GABA_A_ and/or GABA_B_ receptors, GABA induces postsynaptic neuronal hyperpolarization. But neuron-released GABA can also act on the GABA receptors of OPCs modulating axonal myelination. In addition, extrasynaptic GABA is taken up by neuronal GAT1 and astroglial GAT3 transporters. Both transporters, however, are also expressed by OPCs, but functional studies are still required to determine their roles. **C** Also, OLs can express GS to produce glutamine. The latter might be transported to myelinated axons, where it can be converted into GABA. Additional experiments are still required to test this hypothesis.

GABA availability in the CNS is either ensured by synthesis from glutamate by the glutamic acid decarboxylase enzymes (GAD) 67 and GAD65 [27, 28] or by monoacetylation of putrescine [29, 30]. Synthesis by GADs in the glutamine-glutamate cycle (GGC) is the most common pathway and GABA level are mostly determined by the activity of GADs. Briefly, in the GGC, glutamate is transformed into glutamine by glutamine synthetase of astrocytes (Fig. 1A, B). Glutamine is released by several types of glutamine transporter and taken up by neurons, where it is converted into glutamate. The latter is finally processed by GADs to produce GABA in GABAergic neurons [31] (Fig. 1B). Although GAD67 and GAD65 share a large similarity of their genes (*GAD1* and *GAD2*, respectively), their expression pattern and functions are quite disparate. GAD67 is uniformly distributed in the whole cell while GAD65 is mainly found in the axonal terminals [32]. In addition, GAD67 is already expressed during early development while GAD65 is more prominent in later stages (reviewed by [27]). These spatial and temporal differences are highly related to their functions. GABA produced by GAD67 mainly functions as a neurotrophic factor and is independent of neurotransmission, e.g., involved in synaptogenesis during development (reviewed by [27]). GAD65, however, is responsible for synaptic neurotransmission. Therefore, it is not surprising that GAD67-null mice cannot survive longer than a day after birth, while GAD65-null mice are born with slowly developing spontaneous seizures [33, 34]. Although these deficits are highly likely attributable to disordered neuronal GABA synthesis, the GABA contribution from glial cells must not be neglected. GAD65 and GAD67 are both expressed in glial cells [35]. Astrocytes of the olfactory bulb, hippocampus, thalamus, and cerebellum (i.e., Bergmann glia) release GABA to inhibit neighboring neuronal activity [36–39]. Recently, GAD65/67 and monoamine oxidase B, as well as GABA were found in OPCs and oligodendrocytes *in vitro* [40]. These findings suggest the potential of autocrine or paracrine GABAergic signaling pathways for oligodendrocyte (OL) development and/or neural circuit formation. Besides astrocytes, OLs also express glutamine synthetase in caudal regions and the spinal cord [41], providing a potential source of glutamine for axons *via* myelin-axon communication (Fig. 1A, C). In the case of inhibitory axons, glutamine is further transformed into GABA (Fig. 1C). More studies are required to confirm the functional GABA synthesis, release, and uptake in cells of the OL lineage.

GABA-containing transmitter vesicles (vGAT) are filled in synaptic terminals (Fig. 1B) and released in a Ca^2+^-dependent manner. The general mechanism of vesicular exocytosis, membrane fusion, and release of anchored GABA vesicles is triggered by Ca^2+^ influx through voltage-gated Ca^2+^ channels (VGCCs). In addition, GABA can reach the extracellular space *via* reversal of GABA transporters (GATs), called non-vesicular release [42–44]. Previously, GATs were mainly considered to be responsible for GABA uptake from the synaptic cleft. For this GABA uptake, GATs utilize the chemical Na^+^ gradient, aided by a Cl^−^ gradient; e.g., neuronal GAT1 co-transports two Na^+^ and one Cl^−^ together with one GABA molecule. This transport not only increases the intracellular levels of GABA, Na^+^, and Cl^−^, it also depolarizes the neuron. Under baseline conditions, GATs operate near equilibrium [43]. Therefore, upon moderate depolarization evoked by a short series of action potentials, transporter reversal occurs [45, 46]. However, during excessive network activity and enhanced synaptic GABA release, elevated levels of extracellular GABA favor GABA uptake by GATs [47]. Therefore, how the operation of GATs, including their reversal, is exactly controlled and how this process is related to physiological functions is yet unclear.

As a very complex but highly precise organ, our brain keeps a balance of excitatory and inhibitory signals to control proper behavioral performance. As reported, both vGAT-null (little, if any, GABA release) [48] and GAT1-null (no GABA clearance) mice cannot survive beyond birth [49]. Therefore, it is critical to maintain GABA homeostasis in the extracellular space by synchronized regulation of GABA release and uptake. In the brain, two different GATs fine-tune the neuronal excitability: GAT1 (*SLC6A1*) on presynaptic terminals and GAT3 (*SLC6A11*) on perisynaptic astroglial processes (Fig. 1B). Transcriptome studies have revealed that astroglial GAT3 dominates over GAT1. In addition to neurons, OPCs and OLs express functional GAT1, though at rather low levels [35, 40, 50] (Fig. 1B). However, functional studies demonstrating the biological impact of GAT1 for cells of the OL lineage are still missing. In addition to GAT1 and 3, some GAT2 (*SLC6A13*) immunoreactivity has been observed on CNS blood vessels [51]. GAT2 mainly permits efflux of GABA and taurine from the brain to the circulating blood stream [51]. Therefore, GAT2-deficient mice have slightly increased taurine in the brain [52]; however, they perform normally under physiological conditions. Transcriptome data suggest GAT2 expression by OPCs, though at a low level. This is interesting in respect to the current notion that OPCs can also contribute to the blood-brain barrier (BBB) while migrating along blood vessels during development [53]. Taken together, these findings suggest a potential novel function of OPCs in neural circuits, by either taking up GABA from extracellular space or by being associated with the overall GABA efflux through the BBB to the periphery. Nevertheless, more functional studies are required to identify the role of GAT2 in OPCs. In juvenile rats, GAT1 and GAT3 have also been detected in OLs [40], however, it is yet elusive whether and how both GATs function in OL GABA circulation.

## GABA Receptors and Their Biological Actions on Neurons

**Fig. 2:**
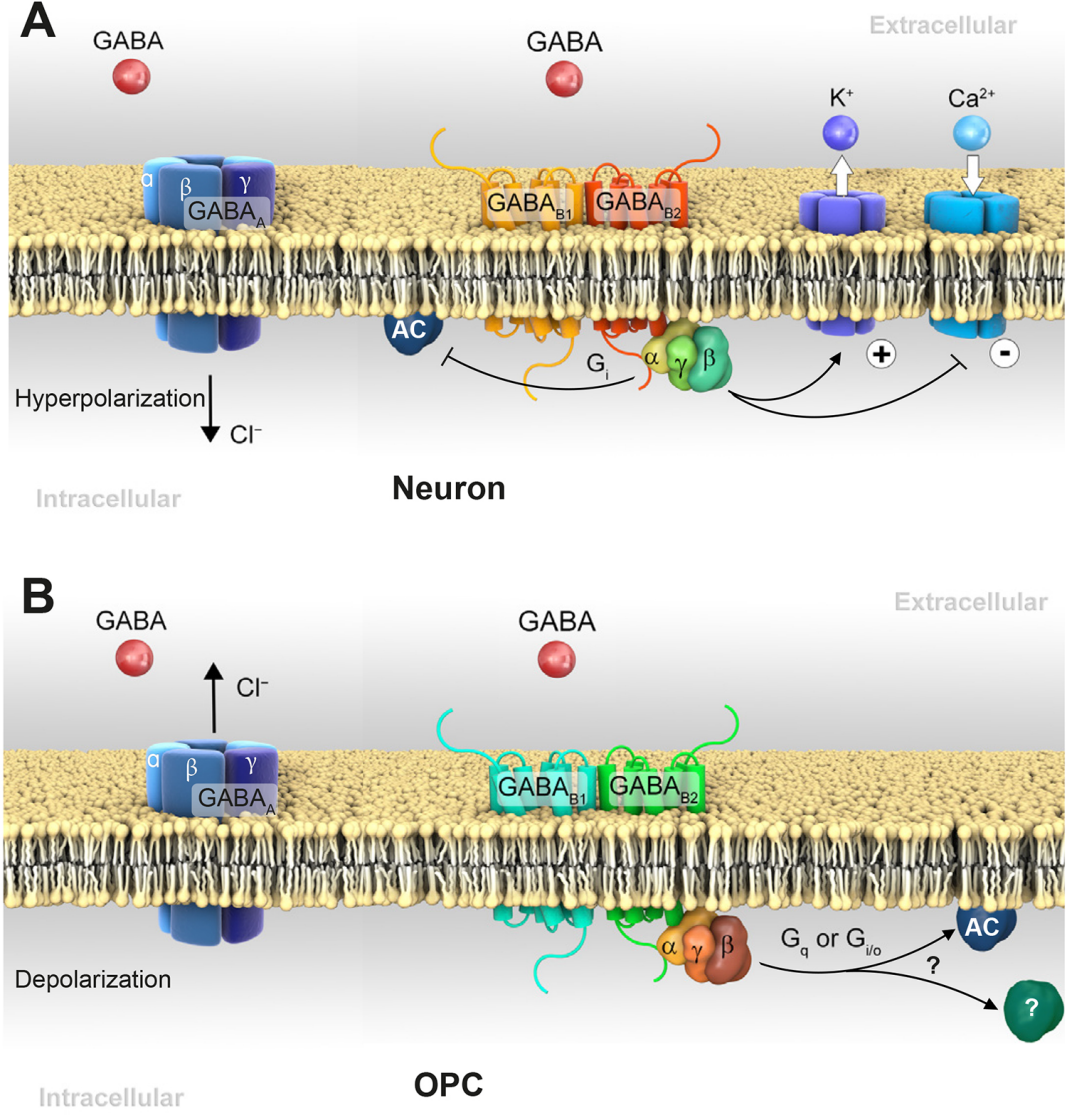
GABA receptor expression in neurons and OPCs. **A** Activation of ionotropic GABA_A_ receptors induces Cl^−^ influx to hyperpolarize neurons. The GABA_B1_ subunit confers ligand-binding, while the B2 subunit transduces the GABA signal into the cell. Activation of the neuronal GABA_B_ receptor induces dissociation of G_α_ and G_βγ_ subunits. The G_α_ subunit inhibits adenylyl cyclase (AC), while G_βγ_ activates G protein-gated inwardly rectifying K^+^ channels and inhibits voltage-gated Ca^2+^ channels (VGCCs), thereby reducing neurotransmitter release. The regulation of VGCCs can occur pre- and postsynaptically. **B** Different from neurons, in OPCs, activation of GABA_A_ receptors causes a Cl^−^ efflux and depolarization based on the higher levels of cytosolic Cl^−^. GABA_B_ receptors expressed in OPCs are thought to transduce signals *via* G_α_ with or without association of G_βγ_; or *via* the G_q_ pathway linked to phospholipase C, further increasing intracellular Ca^2+^ release from the endoplasmic reticulum.

To exert inhibition, GABA binds to two distinct receptors: GABA_A_ and GABA_B_. GABA_A_ receptors are ligand-gated ionotropic transmembrane receptors, permeating Cl^–^ ions in both directions [54]. To date, a plethora of 19 GABA_A_ receptor subunits have been identified in the mammalian CNS: α1–6, β1–3, γ1–3, δ, ɛ, θ, π, and ρ1–3 [55]. In general, the pentameric receptor assembly is composed of two α, two β and one γ subunit (Fig. 2A, B). Due to various subunit compositions and distinct regional distributions, GABA_A_ receptors exhibit tremendous diversity in terms of biophysical properties and dynamic regulation [55, 56]. Since the subunits ρ1–3 form complexes with themselves only, and not with other subunits, they are designated as GABA_C_ or GABA_A-ρ_ receptors. However, they are similar to GABA_A_ receptors in structure, function, and mechanism of action [57].

The GABA_A_ receptor is permeable to Cl^−^ anions in both directions depending on the difference between extra- and intracellular Cl^−^ concentrations. In general, extracellular Cl^−^ is above its equilibrium potential. Therefore, upon postsynaptic GABA_A_ receptor activation, a fast Cl^−^ influx generates neuronal hyperpolarization. This raises the threshold for postsynaptic action potentials and thereby decreases excitatory neurotransmitter release, i.e., inhibitory neurotransmission [58, 59] (Fig. 2A). Notably, GABA_A_ receptors are also expressed at extra-synaptic regions. These receptors can be activated by GABA spillover, leading to tonic inhibition [55].

GABA_B_ receptors are metabotropic G-protein-coupled receptors. Two major GABA_B_ receptor isoforms (GABA_B1_ and GABA_B2_) and various splice variants (GABA_B1a–g_) have been described [60, 61]. GABA_B1_ and GABA_B2_ are co-expressed, generating functional receptors in a heterodimeric assembly [62–64], although some functional homodimers have been described as well [65]. The ligand-binding B1 subunit remains in the endoplasmic reticulum through a retention signal until assembly with the B2 subunit [66]. Only the assembled receptor dimers reach the cell surface and function. GABA activation occurs *via* a Venus flytrap domain of the B1 subunit [60, 67].

Neuronal GABA_B_ receptors are located in both pre- and postsynaptic membranes. Its G protein activation triggers dissociation of G_α_ and G_βγ_ subunits. Binding of G_βγ_ to VGCCs leads to reduced presynaptic Ca^2+^ influx preventing vesicular release (Fig. 2A) [68, 69], while decreased postsynaptic Ca^2+^ current suppresses neuronal excitability [70, 71]. In addition, postsynaptically, G_αi/o_ inhibits adenylyl cyclase, thereby reducing cAMP levels, while G_βγ_ activates G protein-gated inwardly-rectifying K^+^ channels, hyperpolarizing the postsynaptic membrane (Fig. 2A). GABA_B_ receptors regulate gene expression by interacting with activating transcription factor 4 (ATF-4), a member of the cAMP response element-binding protein (CREB)/ATF family [60, 72, 73]. Disruption of GABA_B_ receptor-mediated responses has been associated with several neuropathologies including epilepsy and hyperalgesia [74].

Apart from acting as an inhibitory neurotransmitter, GABA is also considered to be a neurotrophic factor. In cultured cerebellar granule cells, retinal neurons, and neuroblastoma neurons, GABA promotes neurite growth [75]. Another peculiar finding is that GABA can act as an excitatory neurotransmitter in cortical and hippocampal neurons during early postnatal days [76–78]. At this age, the Nernst potential of Cl^−^ is positive in respect to the resting membrane potential due to higher activity of the cation-chloride importer Na-K-Cl cotransporter in comparison to the extruder K^+^-Cl^−^ cotransporter 2, and the opening of GABA_A_ receptors results in Cl^−^ efflux with subsequent depolarization [79].

## Expression of GABA Receptors in Cells of the Oligodendrocyte Lineage

Already in 1984, GABA-evoked responses were reported in a subpopulation of OLs from explant cultures of the mouse spinal cord [54]. These cells were depolarized by GABA (1 mmol/L, 4 mV depolarization). This depolarization was sensitive to competitive as well as non-competitive GABA_A_ receptor antagonists [54]. These experiments provided the first evidence of the functional expression of GABA_A_ receptors in OLs. A follow-up study on cultured OPCs and OLs further demonstrated that the GABA-induced depolarization (10^−2^ mmol/L, 30–680 pA in 60% of the OL lineage cells) was due to Cl^−^ efflux [80] (Fig. 2B). Also, in acutely isolated slices of corpus callosum and hippocampus, GABA_A_ receptors evoked depolarization in OPCs (1 mmol/L GABA, 75 pA and 324 pA, respectively) [81, 82]. Notably, GABA_A_ receptor expression was found to be down-regulated during the lineage progression from proliferating OPCs to myelinating OLs. The current response to GABA as well as intracellular Ca^2+^ increases were drastically reduced *in situ* [80, 81, 83] and *in vitro* [84]. In line with this, recent transcriptome studies as well as single-cell qRT-PCR have shown a decrease of all GABA_A_ receptor subunits (α1–5, β1–3, and γ1–3) through OL development [35, 85, 86]. In particular, the γ2 subunit is only expressed in OPCs and not in OLs [35, 85, 86]. Interestingly, the γ2 subunit is specifically detected at the postsynaptic OPC membranes of parvalbumin fast-spiking interneuron-OPC synapses [87], at levels comparable to neuronal postsynaptic expression [88, 89]. Of note, the γ2 subunit is required for the postsynaptic clustering of GABA_A_ receptor subunits [88]. From postnatal week 2 to 4, the number of OPCs expressing α2, α5, β1, and γ2 is decreased while that of α3 and 4 is increased [86]. Of interest, this is the exact age when the synaptic transmission of OPCs switches to extra-synaptic communication [20]. However, the γ2 subunit does not appear to affect OPC proliferation and differentiation, which appears unperturbed in mice with conditional deletion of the γ2 subunit in OPCs [90].

While GABA_A_ receptor levels are strongly reduced in mature OLs [35, 80, 83, 84], axonal contacts trigger the expression of α1 and α3 *in vitro* as well as *in situ* [83]. However, neuronal activity does not appear to be required, since blocking it with tetrodotoxin did not alter the OL response to GABA in neuron-OL co-cultures. It is not clear yet whether these two subunits co-assemble in the same GABA_A_ receptor complex or whether they are components of separate and distinct receptors. Additional studies are required to address the functional role of α1 and α3, but also of other GABA_A_ receptor subunits in OPCs and OLs.

It will be exciting to learn how the spatial-temporal pattern of each subunit, including its subcellular localization, can be correlated with distinct functions in the various subpopulations of the OL lineage. The heterogeneity of OLs, in terms of anatomical location in the brain, was already described at 1921 by del Río Hortega [91]. A century later, using the single-cell RNAseq approach, studies have provided direct evidence for and confirmed an even more complex heterogeneity of OL lineage cells [92–94]. Reconsidering the early finding that only a subpopulation of OLs respond to GABA [54], we are now confronted with numerous subgroups of OLs that may or may not express GABA receptors. And, even if they are expressed, the pentameric composition of each receptor might differ in each subgroup and result in a huge diversity of GABA responses. So far, it is too early to speculate about the exact role of each subunit.

The metabotropic GABA receptor subunits GABA_B1_ and GABA_B2_ are both expressed throughout the OL lineage [35], from the subventricular zone [95] to the corpus callosum [40] and spinal cord [65]. However, so far, GABA_B_ receptors have not been detected in compact myelin structures [96]. Both B1 and B2 subunits were found to be down-regulated during OPC differentiation to OLs *in vitro* [95]. Intriguingly, the ratio of GABA_B1_ to GABA_B2_ also changes with the differentiation of OPCs into OLs, suggesting that B1 or B2 subunits can cooperate with other elements, even forming homodimers with novel functions as is known for some neurons [97, 98]. In the hippocampus of GABA_B2_-null mice, an atypical electrophysiological GABA_B_ response has been recorded, suggesting that GABA_B2_ is not indispensable for GABA_B_ receptor signaling [97]. In addition, several studies also reported coupling of the GABA_B2_ subunit with other G-protein-coupled, heptahelical receptors. The GABA_B2_ subunit is functionally paired with the M2 muscarinic receptor in cortical neurons [98]. As well, functional cooperation of GABA_B2_ subunits and somatostatin receptor 4 has been found in the non-perisynaptic processes of astrocytes [99]. All these reports point to close interactions of GABA_B_ receptor subunits with other G-protein-coupled receptors. However, additional studies are necessary to determine whether this applies to OPCs and/or OLs and if this might change with aging.

## Physiological Functions of GABA Receptors in the Lineage of Oligodendrocyte

### Proliferation, Differentiation, and Myelination

While the sensitivity to GABA is largely reduced in mature OLs [65, 81, 95], a pivotal role of GABA signaling has been suggested during the origin of OPCs and the initial stages of axon recognition and myelination [22, 100]. Systemic application of the GABA_A_ receptor antagonist bicuculline drastically increased OPC proliferation while an increase of GABA evoked the opposite in cerebellar white matter [22]. In addition, endogenous GABA bisected the number of OPCs and mature OLs in organotypic slice cultures of mouse cortex, and this was reversed by the GABA_A_ receptor blocker GABAzine [18], suggesting an inhibitory role of GABA_A_ receptor signaling on OPC self-renewal and myelination [18]. However, it is still elusive whether this occurs by direct activation of OPC GABA_A_ receptors or by a more complex process integrating the activation of OPC GABA_A_ receptors and signals from a GABA-evoked neuronal response.

**Fig. 3:**
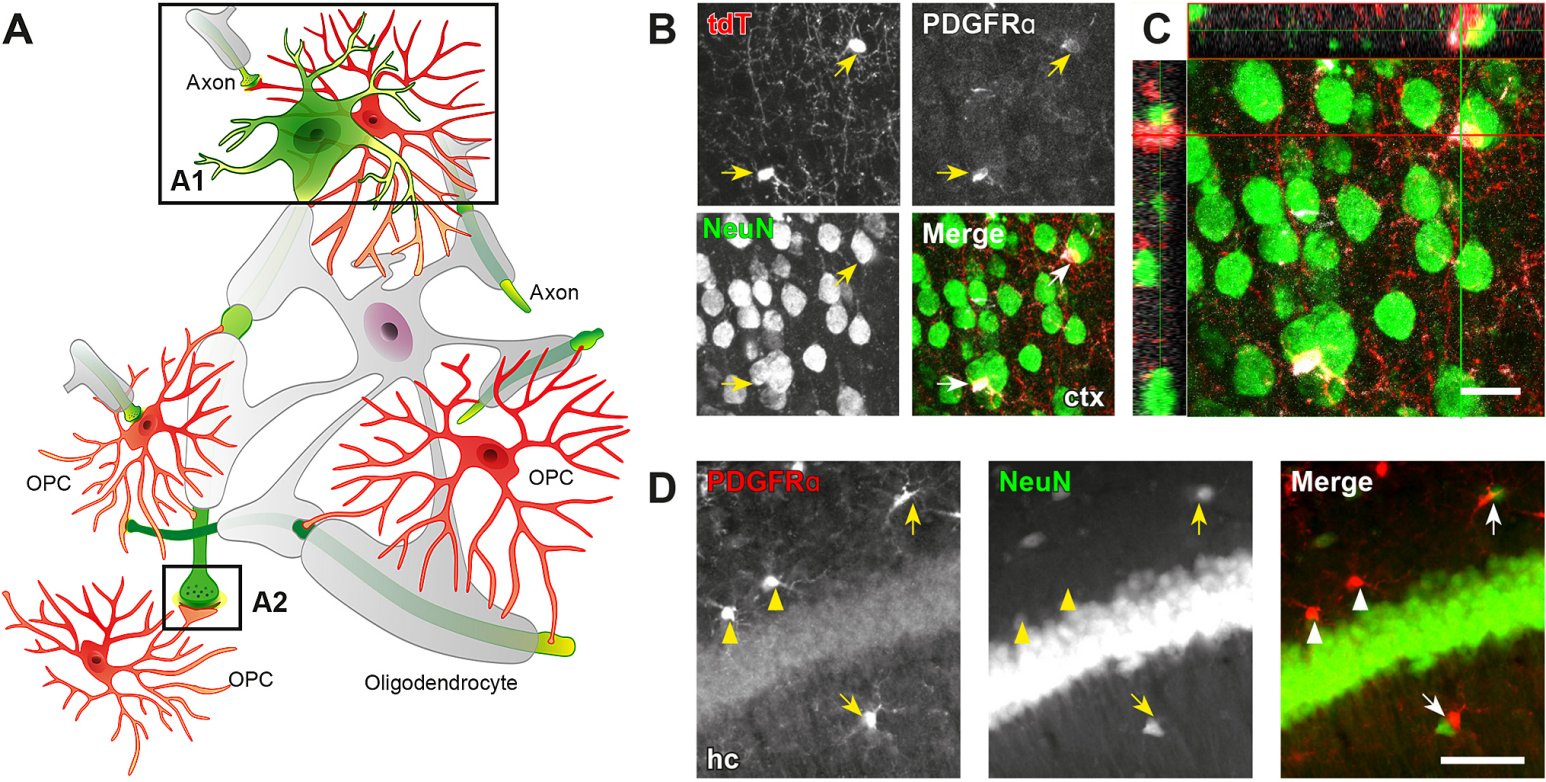
Synaptic and non-synaptic neuron-OPC communication. **A** Schematic of neuron-OPC communication in the brain, including direct soma-soma (**A1**) and synaptic contact (**A2**). **B–D** OPC somata (PDGFRα^+^, red) are in close contact with neuronal somata (NeuN^+^, green) (arrows) in cortex (ctx, **B** and **C**) and hippocampus (hc, **D**). Micrographs in **B** and **C** are from the cortex of NG2-CreER^T2^ × Rosa26-CAG-lsl-tdTomato mice [6, 133]. Images were acquired by confocal laser-scanning (LSM710, **B** and **C**) or automated epifluorescence microscopy (AxioScan.Z1) (**D**) with appropriate filters and objectives. Scale bars, 20 μm for **B** and 50 µm for **D**.

GABAergic signaling of the OL lineage seems to be essential for interneuron myelination. First of all, in layers 2/3 and 4 of cortex, the majority of myelinated axons are interneurons [26]. Among these, parvalbumin (PV)-positive interneurons account for a large proportion. Secondly, interneuron myelination is positively related to axonal activity and caliber [24, 25]. Considering that PV neurons are fast-spiking interneurons in the neocortex [101, 102], these studies strongly suggest a putative GABAergic communication between PV interneurons and OPCs. Indeed, a recent study revealed that disruption of PV interneuron-OPC interaction due to a loss of the γ2 subunit of GABA_A_ receptors in OPCs results in hypomyelination of PV neurons in the barrel cortex [103]. PV-OPC synaptic structures were visualized by Tanaka *et al.* in 2009 [104]. A few years earlier, interneuron-OPC synapses were first detected in acute hippocampal slice preparations by Lin and Bergles [105]. CA1 interneurons directly release GABA, acting on the postsynaptic GABA_A_ receptors of OPCs. These inhibitory neuron-OPC synaptic structures have been subsequently confirmed in numerous studies [20–22, 104] in both grey and white matter [20–22, 86, 87, 90, 105, 106] (Fig. 3A). In cortex, for instance, OPC synapses are ~90% inhibitory [87]. This synaptic transmission (*via* GABA_A_ receptors) peaks at the second postnatal week (p10), and is immediately followed by a drastic increase in the OL population [20]. However, the communication pattern switches to extra-synaptic until the fourth postnatal week, when the GABAergic currents of OPCs are mainly elicited by GABA spillover. Of note, at this time point, the differentiation of cortical OLs is largely completed, further suggesting that, in the early postnatal cortex, synaptic interneuron-OPC contacts are essential for OPC differentiation and interneuron myelination. Extra-synaptic GABA level, however, could be involved in the adaptive regulation of myelination. Indeed, forced increases of GABAergic connectivity between interneurons and first-wave OPCs favor deep layer myelination in the somatosensory cortex [106]. It will be interesting to investigate whether different waves of OPCs [107] form synapses with impact on distinct neuronal network activity or other biological processes. In addition, it is important to state that GABA-mediated myelination might be very different from glutamate-based processes, as indicated by shortened nodes and internodes as well as higher myelin basic protein expression of myelinated GABAergic axons than in non-GABAergic axons [26].

To date, no direct evidence is available demonstrating a decisive role of GABAergic signaling for the development of OL lineage cells *in vivo*. *In vitro*, GABA application fails to affect primary OPC proliferation [108, 109], while selective activation of GABA_B_ receptors with baclofen promotes the proliferation of the OPC cell line CG-4 [95]. These results further suggest the manifold roles of GABA when activating both GABA_A_ and GABA_B_ receptors leading to a complex series of events. However, the expression and even the functions of GABA receptors could differ between primary OPCs and stable cell lines. Indeed, a recent *in vitro* study showed that GABA_B_ receptor activation favors primary OPC differentiation rather than self-renewal and survival [40]. Nevertheless, an *in vivo* investigation is necessary to clarify the exact biological function of GABA receptors. In fact, the conditional knockout of the GABA_A_ receptor γ2 subunit during early development (p3–p5) does not influence OPC proliferation and differentiation [90]. Absence of the γ2 subunit reduces the number of OPCs without affecting differentiation into OLs, suggesting that γ2-mediated interneuron-to-OPC synapses might be required for the fine tuning of OPC self-maintenance [90].

## Migration

OPCs maintain their density while migrating to either their target areas followed by differentiation or into sites of injury where they contribute to scar formation [9]. The migration is partially modulated by GABAergic signaling [95, 110], as has been shown for isolated primary OPCs and OPCs in explant preparations. Furthermore, this impact on migration appears to be more dominated by GABA_A_ than GABA_B_ receptor signaling, since it is blocked by the GABA_A_ antagonist bicuculline, but not affected by GABA_B_ antagonists [110]. However, GABA_B_ receptors have been found to promote the migration of CG-4 cells [95]. Again, such differences might be due to the distinct properties of OPCs *in vivo* versus *in vitro* and changes in stable cell lines. Receptor expression as well as the ratio of GABA_A_/GABA_B_ receptors might change during the isolation and culturing processes. And most importantly, the microenvironment, i.e., the three-dimensional tissue organization including the stiffness and composition of the extracellular space, strongly influences migration. Therefore, *in vivo* studies are inevitably needed to address the impact of GABAergic signaling on OPC migration.

## Monitoring Network Activity

OPCs receive GABAergic input in two non-exclusive modes, either directly *via* neuron-OPC synapses, i.e., contact sites between OPC processes and neuronal compartments including nodes of Ranvier, or, more diffusely, from GABA spillover from adjacent neuron-neuron synapses [20]. Close contacts between neuronal somata and OPCs have also been observed, although neurotransmitter-based connectivity is absent at such locations [111, 112] (Fig. 3A–D). About 40% of all cortical OPCs are in close contact with ~4% of all cortical neurons, and these are mostly GABAergic. These anatomically close pairs of neurons and OPCs do not communicate *via* synaptic structures. However, these cell-cell contacts could very well monitor neural network activity [113], similar to the way astrocytes sense their adjacent environment [114]. In the hippocampus, the pairs of OPCs and neurons can receive the same synaptic input from another neuron. OPCs closely apposed to neurons exhibit strongly synchronized excitatory postsynaptic currents [111]. Interestingly, in the cortex, such anatomical proximity is increased when mice are treated with the GABA_B_ receptor agonist baclofen or the GABA_A_ receptor antagonist picrotoxin. OPCs can sense presynaptic excitatory signals after positioning their soma and synapse close to interneurons and thereby regulate the local network. Considering the heterogeneity of OPCs [115], it is also possible that a certain subpopulation of OPCs favors this soma-soma communication. However, more *in vivo* experiments are necessary to address the cause and importance of such contacts.

## Signaling Pathways of GABA Receptors in the OL Lineage

In OPCs, the activation of GABA_A_ receptors induces membrane depolarization *via* Cl^−^ efflux. Concomitantly, AMPA (α-amino-3-hydroxy-5-methyl-4-isoxazolepropionic acid)-type glutamate receptor currents are inhibited [105]. The activation of GABA_A_ receptors also raises the intracellular Ca^2+^ concentration [20, 84, 104, 116, 117] *via* at least two distinct pathways. (1) GABA-induced depolarization activates voltage-gated Na^+^ channels expressed by OPCs. Subsequently, increases of intracellular Na^+^ reverses the activity of the Na^+^-Ca^2+^ exchanger and causes Ca^2+^ elevation in OPCs. This Ca^2+^ signaling pathway, without using VGCCs, is involved in the migration of OPCs [110]. (2) In the adult mouse cortex, GABA-evoked depolarization activates VGCCs, thereby directly elevating [Ca^2+^]_i_. and promoting the release of BDNF (brain derived neurotrophic factor) in the sensory-motor area and entorhinal cortex [104].

In contrast, the activation of GABA_B_ receptors negatively regulates adenylyl cyclase *via* G_αi/o_ proteins and dampens the intracellular cAMP levels of OPCs [95]. Subsequently reduced protein kinase A activity suppresses gene transcription for BDNF and AMPA receptors *via* altered phosphorylation and the nuclear translocation of transcription factors such as CREB protein, thereby modulating synaptic and neural plasticity [118–120]. In cultured OPCs, GABA_B_ receptor-mediated differentiation has also been shown to involve Src-family kinases, which are known to be associated with myelination [40]. Again, additional *in vivo* studies need to be carried out to elucidate the exact downstream pathways of OL GABA_B_ receptors (G_αi/o_ and/or G_q_) and the potential involvement of cAMP and/or Ca^2+^ (Fig. 2B).

## GABA Signaling Under Pathological Conditions

As the major inhibitory neurotransmitter in the brain, GABA plays crucial roles not only in physiological processes but also in many neurological disorders [121, 122]. To date, disturbances of GABAergic signaling have been robustly studied, but significantly less is known for the cells of the OL lineage.

In hypoxic regions associated with a stroke insult, GABA release is drastically increased at the penumbra [123, 124]. Counterintuitively, the GABA_A_ receptor-mediated synaptic input to OPCs is reduced [22], but accompanied by extensive proliferation of OPCs, delayed OL maturation, and abnormal myelination [22]. This coincides with the finding that under physiological conditions GABA acts as neurotrophic factor. GABA *via* GABA_A_ (at least γ2 subunit) receptors does not influence OPC proliferation and myelination [108], while GABA_B_ receptor activation promotes myelination, at least *in vitro* [40], suggesting an inhibitory function of GABA_A_ receptors in myelination. However, whether this communication is synaptic or extrasynaptic is unclear. Upon GABAergic stimulation, adult cortical OPCs produce neurotrophic factors like BDNF, which are increased after stroke [104]. BDNF, in turn, promotes OPC proliferation under physiological and pathological conditions [13, 14]. Whether the newly generated OPCs participate in the regeneration is unknown.

In a rat model of temporal lobe epilepsy, GABA-mediated inhibition is reduced due to two processes: (1) GABA synthesis is decreased mainly due to decreased GAD65 levels and (2) inhibitory postsynaptic currents (IPSCs) decline because of down-regulation of GABA_A_ (especially subunits α1, γ, and δ) and GABA_B_ receptors. However, GABA_A-α5_ and CREB are up-regulated [125]. As an effector of CREB, BDNF expression is increased by seizure activity, which in turn induces hyperexcitability in hippocampal neurons [126]. In mice with mutant CREB, epilepsy is suppressed, suggesting a potential therapeutic option to target epilepsy [127]. However, whether and how GABA_A_ and GABA_B_ receptor-CREB signaling pathways in OPCs and OLs also contribute to epileptogenesis needs further analysis.

Dysfunction of GABA-mediated OPC neurotransmission has not yet been demonstrated in multiple sclerosis (MS), a disease with progressive demyelination. But several reports suggest the importance of GABAergic signaling during the course of MS. In the brain of MS patients, both pre- and postsynaptic GABAergic neurotransmission are decreased [128, 129]. However, GABA level are increased in the sensorimotor cortex of MS patients but decreased in the hippocampus [130, 131]. With the knowledge that both GABA_A_ and GABA_B_ receptors are involved in OPC proliferation and differentiation under physiological conditions [18, 95], GABAergic neurotransmission of OPCs and OLs could also affect the disease progression of MS. Indeed, a recent single-cell RNAseq transcriptome study of mature OLs prepared from experimental autoimmune encephalomyelitis (EAE) mice revealed reduced levels of the GABA_B1_ subunit, but unchanged levels of the GABA_B2_ and GABA_A_ receptor subunits [132]. As under physiological conditions, GABA_B_ receptors of OLs also influence myelination in EAE. Interestingly, in these EAE mice, the expression of GABA transporter GAT3 is down-regulated in OPCs, while GAT1 is increased in OLs. However, the mRNA level of the transporter might not coincide with the respective transport activity. Therefore, elevations or reductions of extracellular GABA level cannot be inferred readily. In addition, under pathological conditions, GATs can reverse-transport GABA to the extracellular space. The scenario gets even more complex in light of the according timeline: Are expression changes of GATs a result of demyelination and thereby ahead of the remyelination failure or rather a consequence? Answering how GABAergic signaling in cells of the OL lineage is involved in de- and remyelination remains for the future.

## Conclusion

GABA, a neurotransmitter as well as a neurotrophic factor, is synthetized and taken up by OPCs and OLs. For a long time, GABA has been recognized as the main mediator of neuronal inhibition. Now, we have learnt that this transmitter is broadly sensed by the OL linage, i.e., OL precursor cells as well as mature OLs. In contrast to neurons, however, in OPCs and OLs, GABA positively stimulates signaling cascades, mainly leading to enhanced Ca^2+^ levels. Thereby, GABA promotes myelination as well as neural recovery. GABAergic signaling in cells of the OL lineage cells represents an exciting novel field of research, especially the GABA-dependent interneuron-OPC communication. The concomitant analysis of OL differentiation and the modulation of neuronal network activity by distinct patterns of myelination will not only help to understand the normal brain but will be pivotal in complex neuropathologies that depend on temporally precise neuronal firing and transmission.

## References

[1] Lee SE, Lee Y, Lee GH (2019). The regulation of glutamic acid decarboxylases in GABA neurotransmission in the brain. Arch Pharm Res.

[2] Roberts E, Frankel S (1950). gamma-Aminobutyric acid in brain: its formation from glutamic acid. J Biol Chem.

[3] Krnjević K, Schwartz S (1967). The action of gamma-aminobutyric acid on cortical neurones. Exp Brain Res.

[4] Hösli L, Andrès PF, Hösli E (1978). Neuron-glia interactions: indirect effect of GABA on cultured glial cells. Exp Brain Res.

[5] Nishiyama A, Komitova M, Suzuki R, Zhu X (2009). Polydendrocytes (NG2 cells): multifunctional cells with lineage plasticity. Nat Rev Neurosci.

[6] Huang W, Zhao N, Bai X, Karram K, Trotter J, Goebbels S (2014). Novel NG2-CreERT2 knock-in mice demonstrate heterogeneous differentiation potential of NG2 glia during development. Glia.

[7] Huang W, Bai X, Stopper L, Catalin B, Cartarozzi LP, Scheller A (2018). During development NG2 glial cells of the spinal cord are restricted to the oligodendrocyte lineage, but generate astrocytes upon acute injury. Neuroscience.

[8] Huang W, Guo Q, Bai X, Scheller A, Kirchhoff F (2019). Early embryonic NG2 glia are exclusively gliogenic and do not generate neurons in the brain. Glia.

[9] Hughes EG, Kang SH, Fukaya M, Bergles DE (2013). Oligodendrocyte progenitors balance growth with self-repulsion to achieve homeostasis in the adult brain. Nat Neurosci.

[10] Dimou L, Götz M (2014). Glial cells as progenitors and stem cells: new roles in the healthy and diseased brain. Physiol Rev.

[11] Scheller A, Bai X, Kirchhoff F (2017). The role of the oligodendrocyte lineage in acute brain trauma. Neurochem Res.

[12] Guo Q, Scheller A, Huang W (2021). Progenies of NG2 glia: what do we learn from transgenic mouse models?. Neural Regen Res.

[13] Van't Veer A, Du Y, Fischer TZ, Boetig DR, Wood MR, Dreyfus CF (2009). Brain-derived neurotrophic factor effects on oligodendrocyte progenitors of the basal forebrain are mediated through trkB and the MAP kinase pathway. J Neurosci Res.

[14] Tsiperson V, Huang Y, Bagayogo I, Song Y, VonDran MW, DiCicco-Bloom E*, et al.* Brain-derived neurotrophic factor deficiency restricts proliferation of oligodendrocyte progenitors following cuprizone-induced demyelination. ASN Neuro 2015, 7. 10.1177/1759091414566878. 10.1177/1759091414566878 PMC4720179 25586993

[15] Jiang C, Yang W, Fan Z, Teng P, Mei R, Yang J (2018). AATYK is a novel regulator of oligodendrocyte differentiation and myelination. Neurosci Bull.

[16] Kukley M, Capetillo-Zarate E, Dietrich D (2007). Vesicular glutamate release from axons in white matter. Nat Neurosci.

[17] Kukley M, Kiladze M, Tognatta R, Hans M, Swandulla D, Schramm J (2008). Glial cells are born with synapses. FASEB J.

[18] Hamilton NB, Clarke LE, Arancibia-Carcamo IL, Kougioumtzidou E, Matthey M, Káradóttir R (2017). Endogenous GABA controls oligodendrocyte lineage cell number, myelination, and CNS internode length. Glia.

[19] Bergles DE, Roberts JD, Somogyi P, Jahr CE (2000). Glutamatergic synapses on oligodendrocyte precursor cells in the hippocampus. Nature.

[20] Vélez-Fort M, Maldonado PP, Butt AM, Audinat E, Angulo MC (2010). Postnatal switch from synaptic to extrasynaptic transmission between interneurons and NG2 cells. J Neurosci.

[21] Káradóttir R, Hamilton NB, Bakiri Y, Attwell D (2008). Spiking and nonspiking classes of oligodendrocyte precursor glia in CNS white matter. Nat Neurosci.

[22] Zonouzi M, Scafidi J, Li P, McEllin B, Edwards J, Dupree JL (2015). GABAergic regulation of cerebellar NG2 cell development is altered in perinatal white matter injury. Nat Neurosci.

[23] Ge WP, Yang XJ, Zhang Z, Wang HK, Shen W, Deng QD (2006). Long-term potentiation of neuron-glia synapses mediated by Ca^2+^-permeable AMPA receptors. Science.

[24] Stedehouder J, Couey JJ, Brizee D, Hosseini B, Slotman JA, Dirven CMF (2017). Fast-spiking parvalbumin interneurons are frequently myelinated in the cerebral cortex of mice and humans. Cereb Cortex.

[25] Stedehouder J, Brizee D, Slotman JA, Pascual-Garcia M, Leyrer ML, Bouwen BL*, et al.* Local axonal morphology guides the topography of interneuron myelination in mouse and human neocortex. Elife 2019, 8. 10.7554/eLife.48615. 10.7554/eLife.48615 PMC6927753 31742557

[26] Micheva KD, Wolman D, Mensh BD, Pax E, Buchanan J, Smith SJ*, et al.* A large fraction of neocortical myelin ensheathes axons of local inhibitory neurons. Elife 2016, 5. 10.7554/eLife.15784. 10.7554/eLife.15784 PMC4972537 27383052

[27] Pinal CS, Tobin AJ (1998). Uniqueness and redundancy in GABA production. Perspect Dev Neurobiol.

[28] Deidda G, Bozarth IF, Cancedda L (2014). Modulation of GABAergic transmission in development and neurodevelopmental disorders: investigating physiology and pathology to gain therapeutic perspectives. Front Cell Neurosci.

[29] Petroff OA (2002). GABA and glutamate in the human brain. Neuroscientist.

[30] Yoon BE, Woo J, Lee CJ (2012). Astrocytes as GABA-ergic and GABA-ceptive cells. Neurochem Res.

[31] Hertz L (2013). The glutamate-glutamine (GABA) cycle: importance of late postnatal development and potential reciprocal interactions between biosynthesis and degradation. Front Endocrinol (Lausanne).

[32] Kaufman DL, Houser CR, Tobin AJ (1991). Two forms of the gamma-aminobutyric acid synthetic enzyme glutamate decarboxylase have distinct intraneuronal distributions and cofactor interactions. J Neurochem.

[33] Asada H, Kawamura Y, Maruyama K, Kume H, Ding RG, Kanbara N (1997). Cleft palate and decreased brain gamma-aminobutyric acid in mice lacking the 67-kDa isoform of glutamic acid decarboxylase. Proc Natl Acad Sci U S A.

[34] Kash SF, Johnson RS, Tecott LH, Noebels JL, Mayfield RD, Hanahan D (1997). Epilepsy in mice deficient in the 65-kDa isoform of glutamic acid decarboxylase. Proc Natl Acad Sci U S A.

[35] Zhang Y, Chen K, Sloan SA, Bennett ML, Scholze AR, O'Keeffe S (2014). An RNA-sequencing transcriptome and splicing database of glia, neurons, and vascular cells of the cerebral cortex. J Neurosci.

[36] Kozlov AS, Angulo MC, Audinat E, Charpak S (2006). Target cell-specific modulation of neuronal activity by astrocytes. Proc Natl Acad Sci U S A.

[37] Le Meur K, Mendizabal-Zubiaga J, Grandes P, Audinat E (2012). GABA release by hippocampal astrocytes. Front Comput Neurosci.

[38] Jiménez-González C, Pirttimaki T, Cope DW, Parri HR (2011). Non-neuronal, slow GABA signalling in the ventrobasal thalamus targets δ-subunit-containing GABA_A_ receptors. Eur J Neurosci.

[39] Barakat L, Bordey A (2002). GAT-1 and reversible GABA transport in Bergmann glia in slices. J Neurophysiol.

[40] Serrano-Regal MP, Luengas-Escuza I, Bayón-Cordero L, Ibarra-Aizpurua N, Alberdi E, Pérez-Samartín A (2020). Oligodendrocyte differentiation and myelination is potentiated *via* GABA. Neuroscience.

[41] Xin W, Mironova YA, Shen H, Marino RAM, Waisman A, Lamers WH (2019). Oligodendrocytes support neuronal glutamatergic transmission via expression of glutamine synthetase. Cell Rep.

[42] Attwell D, Barbour B, Szatkowski M (1993). Nonvesicular release of neurotransmitter. Neuron.

[43] Richerson GB, Wu Y (2003). Dynamic equilibrium of neurotransmitter transporters: not just for reuptake anymore. J Neurophysiol.

[44] Schwartz EA (1987). Depolarization without calcium can release gamma-aminobutyric acid from a retinal neuron. Science.

[45] Wu Y, Wang W, Richerson GB (2001). GABA transaminase inhibition induces spontaneous and enhances depolarization-evoked GABA efflux via reversal of the GABA transporter. J Neurosci.

[46] Wu Y, Wang W, Díez-Sampedro A, Richerson GB (2007). Nonvesicular inhibitory neurotransmission via reversal of the GABA transporter GAT-1. Neuron.

[47] Savtchenko L, Megalogeni M, Rusakov DA, Walker MC, Pavlov I (2015). Synaptic GABA release prevents GABA transporter type-1 reversal during excessive network activity. Nat Commun.

[48] Saito K, Kakizaki T, Hayashi R, Nishimaru H, Furukawa T, Nakazato Y (2010). The physiological roles of vesicular GABA transporter during embryonic development: a study using knockout mice. Mol Brain.

[49] Jensen K, Chiu CS, Sokolova I, Lester HA, Mody I (2003). GABA transporter-1 (GAT1)-deficient mice: differential tonic activation of GABA_A_*versus* GABA_B_ receptors in the hippocampus. J Neurophysiol.

[50] Fattorini G, Melone M, Sánchez-Gómez MV, Arellano RO, Bassi S, Matute C (2017). GAT-1 mediated GABA uptake in rat oligodendrocytes. Glia.

[51] Takanaga H, Ohtsuki S, Hosoya Ki, Terasaki T. GAT2/BGT-1 as a system responsible for the transport of gamma-aminobutyric acid at the mouse blood-brain barrier. J Cereb Blood Flow Metab 2001, 21: 1232–1239. 10.1097/00004647-200110000-00012 11598501

[52] Zhou Y, Holmseth S, Guo C, Hassel B, Höfner G, Huitfeldt HS (2012). Deletion of the γ-aminobutyric acid transporter 2 (GAT2 and SLC6A13) gene in mice leads to changes in liver and brain taurine contents. J Biol Chem.

[53] Tsai HH, Niu J, Munji R, Davalos D, Chang J, Zhang H (2016). Oligodendrocyte precursors migrate along vasculature in the developing nervous system. Science.

[54] Gilbert P, Kettenmann H, Schachner M (1984). gamma-Aminobutyric acid directly depolarizes cultured oligodendrocytes. J Neurosci.

[55] Farrant M, Nusser Z (2005). Variations on an inhibitory theme: phasic and tonic activation of GABA_A_ receptors. Nat Rev Neurosci.

[56] Ben-Ari Y, Gaiarsa JL, Tyzio R, Khazipov R (2007). GABA: a pioneer transmitter that excites immature neurons and generates primitive oscillations. Physiol Rev.

[57] Naffaa MM, Hung S, Chebib M, Johnston GAR, Hanrahan JR (2017). GABA-ρ receptors: distinctive functions and molecular pharmacology. Br J Pharmacol.

[58] Kaila K (1994). Ionic basis of GABA_A_ receptor channel function in the nervous system. Prog Neurobiol.

[59] Hübner CA, Holthoff K (2013). Anion transport and GABA signaling. Front Cell Neurosci.

[60] Bettler B, Kaupmann K, Mosbacher J, Gassmann M (2004). Molecular structure and physiological functions of GABA_B_ receptors. Physiol Rev.

[61] Bettler B, Tiao JY (2006). Molecular diversity, trafficking and subcellular localization of GABA_B_ receptors. Pharmacol Ther.

[62] Kaupmann K, Malitschek B, Schuler V, Heid J, Froestl W, Beck P (1998). GABA_B_-receptor subtypes assemble into functional heteromeric complexes. Nature.

[63] Bowery NG, Bettler B, Froestl W, Gallagher JP, Marshall F, Raiteri M*, et al.* International Union of Pharmacology. XXXIII. Mammalian gamma-aminobutyric acid(B) receptors: structure and function. Pharmacol Rev 2002, 54: 247–264. 10.1124/pr.54.2.247 12037141

[64] Kuner R, Köhr G, Grünewald S, Eisenhardt G, Bach A, Kornau HC (1999). Role of heteromer formation in GABA_B_ receptor function. Science.

[65] Calver AR, Medhurst AD, Robbins MJ, Charles KJ, Evans ML, Harrison DC (2000). The expression of GABA_B1_ and GABA_B2_ receptor subunits in the CNS differs from that in peripheral tissues. Neuroscience.

[66] Thuault SJ, Brown JT, Sheardown SA, Jourdain S, Fairfax B, Spencer JP (2004). The GABA_B2_ subunit is critical for the trafficking and function of native GABA_B_ receptors. Biochem Pharmacol.

[67] Galvez T, Parmentier ML, Joly C, Malitschek B, Kaupmann K, Kuhn R (1999). Mutagenesis and modeling of the GABA_B_ receptor extracellular domain support a venus flytrap mechanism for ligand binding. J Biol Chem.

[68] Obrietan K, van den Pol AN (1999). GABA_B_ receptor-mediated regulation of glutamate-activated calcium transients in hypothalamic and cortical neuron development. J Neurophysiol.

[69] Menon-Johansson AS, Berrow N, Dolphin AC (1993). G_o_ transduces GABA_B_-receptor modulation of N-type calcium channels in cultured dorsal root ganglion neurons. Pflugers Arch.

[70] Li Y, Stern JE (2004). Activation of postsynaptic GABA_B_ receptors modulate the firing activity of supraoptic oxytocin and vasopressin neurones: role of calcium channels. J Neuroendocrinol.

[71] Harayama N, Shibuya I, Tanaka K, Kabashima N, Ueta Y, Yamashita H (1998). Inhibition of N- and P/Q-type calcium channels by postsynaptic GABA_B_ receptor activation in rat supraoptic neurones. J Physiol.

[72] Nehring RB, Horikawa HP, El Far O, Kneussel M, Brandstätter JH, Stamm S (2000). The metabotropic GABA_B_ receptor directly interacts with the activating transcription factor 4. J Biol Chem.

[73] White JH, McIllhinney RA, Wise A, Ciruela F, Chan WY, Emson PC (2000). The GABAB receptor interacts directly with the related transcription factors CREB2 and ATFx. Proc Natl Acad Sci U S A.

[74] Schuler V, Lüscher C, Blanchet C, Klix N, Sansig G, Klebs K (2001). Epilepsy, hyperalgesia, impaired memory, and loss of pre- and postsynaptic GABA_B_ responses in mice lacking GABA_B(1)_. Neuron.

[75] Redburn DA, Paul MJ (1987). GABA-its role and development in retina. Progress in retinal research.

[76] Ben-Ari Y, Cherubini E (1991). Zinc and GABA in developing brain. Nature.

[77] Ben-Ari Y, Tseeb V, Raggozzino D, Khazipov R, Gaiarsa JL (1994). gamma-Aminobutyric acid (GABA): a fast excitatory transmitter which may regulate the development of hippocampal neurones in early postnatal life. Prog Brain Res.

[78] Ganguly K, Schinder AF, Wong ST, Poo M (2001). GABA itself promotes the developmental switch of neuronal GABAergic responses from excitation to inhibition. Cell.

[79] Rivera C, Voipio J, Payne JA, Ruusuvuori E, Lahtinen H, Lamsa K (1999). The K^+^/ Cl^−^ co-transporter KCC2 renders GABA hyperpolarizing during neuronal maturation. Nature.

[80] Von Blankenfeld G, Trotter J, Kettenmann H (1991). Expression and developmental regulation of a GABA_A_ receptor in cultured murine cells of the oligodendrocyte lineage. Eur J Neurosci.

[81] Berger T, Walz W, Schnitzer J, Kettenmann H (1992). GABA- and glutamate-activated currents in glial cells of the mouse corpus callosum slice. J Neurosci Res.

[82] Steinhäuser C, Jabs R, Kettenmann H (1994). Properties of GABA and glutamate responses in identified glial cells of the mouse hippocampal slice. Hippocampus.

[83] Arellano RO, Sánchez-Gómez MV, Alberdi E, Canedo-Antelo M, Chara JC, Palomino A (2016). Axon-to-glia interaction regulates GABA_A_ receptor expression in oligodendrocytes. Mol Pharmacol.

[84] Kirchhoff F, Kettenmann H (1992). GABA triggers a [Ca^2+^]_i_ increase in murine precursor cells of the oligodendrocyte lineage. Eur J Neurosci.

[85] Passlick S, Grauer M, Schäfer C, Jabs R, Seifert G, Steinhäuser C (2013). Expression of the γ2-subunit distinguishes synaptic and extrasynaptic GABA_A_ receptors in NG2 cells of the hippocampus. J Neurosci.

[86] Balia M, Vélez-Fort M, Passlick S, Schäfer C, Audinat E, Steinhäuser C (2015). Postnatal down-regulation of the GABA_A_ receptor γ2 subunit in neocortical NG2 cells accompanies synaptic-to-extrasynaptic switch in the GABAergic transmission mode. Cereb Cortex.

[87] Orduz D, Maldonado PP, Balia M, Vélez-Fort M, de Sars V, Yanagawa Y*, et al.* Interneurons and oligodendrocyte progenitors form a structured synaptic network in the developing neocortex. Elife 2015, 4. 10.7554/eLife.06953. 10.7554/eLife.06953 PMC4432226 25902404

[88] Essrich C, Lorez M, Benson JA, Fritschy JM, Lüscher B (1998). Postsynaptic clustering of major GABA_A_ receptor subtypes requires the gamma 2 subunit and gephyrin. Nat Neurosci.

[89] Kneussel M, Betz H (2000). Clustering of inhibitory neurotransmitter receptors at developing postsynaptic sites: the membrane activation model. Trends Neurosci.

[90] Balia M, Benamer N, Angulo MC (2017). A specific GABAergic synapse onto oligodendrocyte precursors does not regulate cortical oligodendrogenesis. Glia.

[91] Del Río Hortega P (1921). La glía de escasas radiaciones (oligodendroglia). Bol Real Soc Esp Hist Nat.

[92] Marques S, Zeisel A, Codeluppi S, van Bruggen D, Mendanha Falcão A, Xiao L (2016). Oligodendrocyte heterogeneity in the mouse juvenile and adult central nervous system. Science.

[93] Marisca R, Hoche T, Agirre E, Hoodless LJ, Barkey W, Auer F (2020). Functionally distinct subgroups of oligodendrocyte precursor cells integrate neural activity and execute myelin formation. Nat Neurosci.

[94] Spitzer SO, Sitnikov S, Kamen Y, Evans KA, Kronenberg-Versteeg D, Dietmann S (2019). Oligodendrocyte progenitor cells become regionally diverse and heterogeneous with age. Neuron.

[95] Luyt K, Slade TP, Dorward JJ, Durant CF, Wu Y, Shigemoto R (2007). Developing oligodendrocytes express functional GABA_B_ receptors that stimulate cell proliferation and migration. J Neurochem.

[96] Charles KJ, Deuchars J, Davies CH, Pangalos MN (2003). GABA_B_ receptor subunit expression in glia. Mol Cell Neurosci.

[97] Gassmann M, Shaban H, Vigot R, Sansig G, Haller C, Barbieri S (2004). Redistribution of GABA_B(1)_ protein and atypical GABA_B_ responses in GABA_B(2)_-deficient mice. J Neurosci.

[98] Boyer SB, Clancy SM, Terunuma M, Revilla-Sanchez R, Thomas SM, Moss SJ (2009). Direct interaction of GABA_B_ receptors with M2 muscarinic receptors enhances muscarinic signaling. J Neurosci.

[99] Mariotti L, Losi G, Lia A, Melone M, Chiavegato A, Gómez-Gonzalo M (2018). Interneuron-specific signaling evokes distinctive somatostatin-mediated responses in adult cortical astrocytes. Nat Commun.

[100] Vélez-Fort M, Audinat E, Angulo MC (2012). Central role of GABA in neuron-glia interactions. Neuroscientist.

[101] Hu H, Gan J, Jonas P. Interneurons. Fast-spiking, parvalbumin+ GABAergic interneurons: from cellular design to microcircuit function. Science 2014, 345: 1255263. 10.1126/science.1255263 25082707

[102] Kawaguchi Y, Katsumaru H, Kosaka T, Heizmann CW, Hama K (1987). Fast spiking cells in rat hippocampus (CA1 region) contain the calcium-binding protein parvalbumin. Brain Res.

[103] Benamer N, Vidal M, Balia M, Angulo MC (2020). Myelination of parvalbumin interneurons shapes the function of cortical sensory inhibitory circuits. Nat Commun.

[104] Tanaka Y, Tozuka Y, Takata T, Shimazu N, Matsumura N, Ohta A (2009). Excitatory GABAergic activation of cortical dividing glial cells. Cereb Cortex.

[105] Lin SC, Bergles DE (2004). Synaptic signaling between GABAergic interneurons and oligodendrocyte precursor cells in the hippocampus. Nat Neurosci.

[106] Orduz D, Benamer N, Ortolani D, Coppola E, Vigier L, Pierani A (2019). Developmental cell death regulates lineage-related interneuron-oligodendroglia functional clusters and oligodendrocyte homeostasis. Nat Commun.

[107] Kessaris N, Fogarty M, Iannarelli P, Grist M, Wegner M, Richardson WD (2006). Competing waves of oligodendrocytes in the forebrain and postnatal elimination of an embryonic lineage. Nat Neurosci.

[108] Gallo V, Zhou JM, McBain CJ, Wright P, Knutson PL, Armstrong RC (1996). Oligodendrocyte progenitor cell proliferation and lineage progression are regulated by glutamate receptor-mediated K^+^ channel block. J Neurosci.

[109] Yuan X, Eisen AM, McBain CJ, Gallo V (1998). A role for glutamate and its receptors in the regulation of oligodendrocyte development in cerebellar tissue slices. Development.

[110] Tong XP, Li XY, Zhou B, Shen W, Zhang ZJ, Xu TL (2009). Ca^2+^ signaling evoked by activation of Na^+^ channels and Na^+^/Ca^2+^ exchangers is required for GABA-induced NG2 cell migration. J Cell Biol.

[111] Mangin JM, Kunze A, Chittajallu R, Gallo V (2008). Satellite NG2 progenitor cells share common glutamatergic inputs with associated interneurons in the mouse dentate gyrus. J Neurosci.

[112] Boulanger JJ, Messier C (2017). Oligodendrocyte progenitor cells are paired with GABA neurons in the mouse dorsal cortex: Unbiased stereological analysis. Neuroscience.

[113] Boulanger JJ, Messier C (2017). Doublecortin in oligodendrocyte precursor cells in the adult mouse brain. Front Neurosci.

[114] Araque A, Carmignoto G, Haydon PG, Oliet SH, Robitaille R, Volterra A (2014). Gliotransmitters travel in time and space. Neuron.

[115] Marques S, van Bruggen D, Vanichkina DP, Floriddia EM, Munguba H, Väremo L (2018). Transcriptional convergence of oligodendrocyte lineage progenitors during development. Dev Cell.

[116] Belachew S, Malgrange B, Rigo JM, Rogister B, Coucke P, Mazy-Servais C (1998). Developmental regulation of neuroligand-induced responses in cultured oligodendroglia. Neuroreport.

[117] Bernstein M, Lyons SA, Möller T, Kettenmann H (1996). Receptor-mediated calcium signalling in glial cells from mouse corpus callosum slices. J Neurosci Res.

[118] Middei S, Houeland G, Cavallucci V, Ammassari-Teule M, D'Amelio M, Marie H (2013). CREB is necessary for synaptic maintenance and learning-induced changes of the AMPA receptor GluA1 subunit. Hippocampus.

[119] Finkbeiner S, Tavazoie SF, Maloratsky A, Jacobs KM, Harris KM, Greenberg ME (1997). CREB: a major mediator of neuronal neurotrophin responses. Neuron.

[120] Tao X, Finkbeiner S, Arnold DB, Shaywitz AJ, Greenberg ME (1998). Ca^2+^ influx regulates BDNF transcription by a CREB family transcription factor-dependent mechanism. Neuron.

[121] Wong CG, Bottiglieri T, Snead OC (2003). GABA, gamma-hydroxybutyric acid, and neurological disease. Ann Neurol.

[122] Chang YY, Gong XW, Gong HQ, Liang PJ, Zhang PM, Lu QC (2018). GABA_A_ receptor activity suppresses the transition from inter-ictal to ictal epileptiform discharges in juvenile mouse hippocampus. Neurosci Bull.

[123] Phillis JW, Smith-Barbour M, Perkins LM, O'Regan MH (1994). Characterization of glutamate, aspartate, and GABA release from ischemic rat cerebral cortex. Brain Res Bull.

[124] Matsumoto K, Lo EH, Pierce AR, Halpern EF, Newcomb R (1996). Secondary elevation of extracellular neurotransmitter amino acids in the reperfusion phase following focal cerebral ischemia. J Cereb Blood Flow Metab.

[125] Mathew J, Balakrishnan S, Antony S, Abraham PM, Paulose CS (2012). Decreased GABA receptor in the cerebral cortex of epileptic rats: effect of Bacopa monnieri and Bacoside-A. J Biomed Sci.

[126] Binder DK, Croll SD, Gall CM, Scharfman HE (2001). BDNF and epilepsy: too much of a good thing?. Trends Neurosci.

[127] Zhu X, Han X, Blendy JA, Porter BE (2012). Decreased CREB levels suppress epilepsy. Neurobiol Dis.

[128] Dutta R, McDonough J, Yin X, Peterson J, Chang A, Torres T (2006). Mitochondrial dysfunction as a cause of axonal degeneration in multiple sclerosis patients. Ann Neurol.

[129] Rossi S, Studer V, Motta C, De Chiara V, Barbieri F, Bernardi G (2012). Inflammation inhibits GABA transmission in multiple sclerosis. Mult Scler.

[130] Cawley N, Solanky BS, Muhlert N, Tur C, Edden RA, Wheeler-Kingshott CA (2015). Reduced gamma-aminobutyric acid concentration is associated with physical disability in progressive multiple sclerosis. Brain.

[131] Bhattacharyya PK, Phillips MD, Stone LA, Bermel RA, Lowe MJ (2013). Sensorimotor cortex gamma-aminobutyric acid concentration correlates with impaired performance in patients with MS. AJNR Am J Neuroradiol.

[132] Falcão AM, van Bruggen D, Marques S, Meijer M, Jäkel S, Agirre E (2018). Disease-specific oligodendrocyte lineage cells arise in multiple sclerosis. Nat Med.

[133] Madisen L, Zwingman TA, Sunkin SM, Oh SW, Zariwala HA, Gu H (2010). A robust and high-throughput Cre reporting and characterization system for the whole mouse brain. Nat Neurosci.

